# Telocytes are the physiological counterpart of inflammatory fibroid polyps and *PDGFRA*‐mutant GISTs

**DOI:** 10.1111/jcmm.13748

**Published:** 2018-08-17

**Authors:** Riccardo Ricci, Maria Cristina Giustiniani, Marco Gessi, Paola Lanza, Federica Castri, Alberto Biondi, Roberto Persiani, Fabio M. Vecchio, Mauro Risio

**Affiliations:** ^1^ Department of Pathology Università Cattolica del Sacro Cuore Fondazione Policlinico Universitario “A. Gemelli” Rome Italy; ^2^ Department of Surgery Università Cattolica del Sacro Cuore Fondazione Policlinico Universitario “A. Gemelli” Rome Italy; ^3^ Department of Pathology Emeritus IRCC Candiolo Italy

**Keywords:** gastrointestinal stromal tumour, inflammatory fibroid polyp, interstitial cell of Cajal, *PDGFRA*‐mutant syndrome, syndromic gastrointestinal stromal tumour, syndromic inflammatory fibroid polyp, telocyte, telocyte hyperplasia, telocytoma, tumour precursor

## Abstract

*PDGFRA* mutations in the gastrointestinal (GI) tract can cause GI stromal tumour (GIST) and inflammatory fibroid polyp (IFP). Hitherto no cell type has been identified as a physiological counterpart of the latter, while interstitial Cajal cells (ICC) are considered the precursor of the former. However, ICC hyperplasia (ICCH), which strongly supports the ICC role in GIST pathogenesis, has been identified in germline *KIT*‐mutant settings but not in *PDGFRA*‐mutant ones, challenging the precursor role of ICC for *PDGFRA*‐driven GISTs. Telocytes are a recently described interstitial cell type, CD34+/PDGFRA+. Formerly considered fibroblasts, they are found in many organs, including the GI tract where they are thought to be involved in neurotransmission. Alongside IFPs and gastric GISTs, GI wall “fibrosis” has been reported in germline *PDGFRA*‐mutants. Taking the opportunity offered by its presence in a germline *PDGFRA*‐mutant individual, we demonstrate that this lesion is sustained by hyperplastic telocytes, constituting the *PDGFRA*‐mutant counterpart of germline *KIT* mutation‐associated ICCH. Moreover, our findings support a pathogenetic relationship between telocyte hyperplasia and both IFPs and *PDGFRA*‐mutant GISTs. We propose the term “telocytoma” for defining IFP, as it conveys both the pathogenetic (neoplastic) and histotypic (“telocytary”) essence of this tumour, unlike IFP, which rather evokes an inflammatory‐hyperplastic lesion.

## 1 INTRODUCTION

Inflammatory fibroid polyps (IFPs) are benign, polypoid lesions usually centred in the submucosa of the gastrointestinal (GI) tract, with a predilection for gastric antrum. They consist of a proliferation of spindle, stellate or epithelioid mesenchymal cells harboured in a vascularized stroma, infiltrated by inflammatory cells (often with numerous eosinophils). Immunohistochemically, the lesional mesenchymal cells express CD34 and platelet‐derived growth factor receptor alpha (PDGFRA). Most IFPs bear *PDGFRA* mutations.^1^, ^2^ A subset of GI stromal tumours (GISTs), accounting for 6%‐7% of the total, also harbours *PDGFRA* mutations, similar to IFPs.^3^ This GIST subgroup, which manifests a strong predilection for the stomach, is frequently at least partly epithelioid and, unlike other GISTs, is often negative or only faintly/patchy positive for CD117.

No physiological counterpart has hitherto been described for IFPs, although it has been suggested that they may develop from PDGFRA‐positive mesenchymal cells found along the villus membrane in the small intestine.^2^, ^4^ Conversely, GISTs as a whole are commonly considered to be related to interstitial cells of Cajal (ICCs).^5^ Beyond the immunophenotype shared between most GISTs and ICCs (CD117+, DOG1+), the presence of ICC hyperplasia (ICCH) both in human germline *KIT*‐mutant kindreds and in mice engineered to express *KIT* mutations found in human GISTs strongly supports a relationship between ICCs and GISTs.^6^, ^7^ However, to date, no ICCH has been detected either in human germline *PDGFRA*‐mutants or in mice generated for the conditional expression of mutant *PDGFRA*, arguing against a relationship between ICCs and *PDGFRA*‐driven GISTs. Conversely, GI stromal cell hyperplasia has been described in these conditions, especially at submucosal level.^7^, ^8^, ^9^, ^10^

Telocytes (TCs) are a type of interstitial cell, formerly named interstitial Cajal‐like cells, described in the connective tissue of several organs, including the GI tract.^11^, ^12^, ^13^, ^14^, ^15^, ^16^, ^17^, ^18^, ^19^, ^20^, ^21^, ^22^, ^23^ For a long time, they went unrecognized and were labelled simplistically as fibroblasts. In the GI tract, TCs are CD34+, PDGFRA+ and CD117‐, and are thought to play a role in neurotransmission, possibly by spreading the low waves generated by ICCs.^24^ A relationship between TCs and GISTs (without genotypic distinction) or IFPs has been sometimes suggested, based on shared immunohistochemical markers and/or, in the former instance, to explain the occurrence of extragastrointestinal cases.^8^, ^25^

We previously published a kindred bearing a germline mutation (CC→TT at 1957‐58) that led to a Leu for Pro substitution at 653 (P653L) in *PDGFRA* gene.^9^ The proband featured GI tumours encompassing GISTs, IFPs (including tumours previously described as “fibrous tumours”^26^) and a lipoma, and an intramural diffuse stromal cell proliferation, heavily affecting the submucosa, in the stomach and proximal duodenum. We carried out a morphological and immunophenotypical investigation of the histotype of these stromal cells in order to establish their possible morphogenetic and/or pathogenetic relationship with synchronous IFPs and GISTs.

## 2 MATERIALS AND METHODS

### 2.1 Tissue specimens

The gastric tissues investigated were from the only member of a previously published germline *PDGFRA*‐mutant kindred who featured a remarkably marked stromal proliferation of the gastroduodenal wall together with IFPs and GISTs.^9^ The procedures followed were in accordance with the ethical standards of the local institutional committee on human experimentation and with the Helsinki declaration of 1975, as revised in 1983. Informed written consent for this study was obtained from the patient.

### 2.2 Histology and immunohistochemistry

Sections from formalin‐fixed, paraffin‐embedded specimens were stained with haematoxylin/eosin. The following antibodies were employed: CD117 (DAKO, Glostrup, Denmark, rabbit polyclonal, 1:400), CD34 (Novocastra, Newcastle, UK, 1:50) and PDGFRA (sc‐338) (Santa Cruz, Santa Cruz, CA, rabbit polyclonal, 1:400) (in all cases for 30′ at room temperature). Antigen retrieval was performed for PDGFRA (10 minutes at 97°C, in 0.01 M citrate buffer, pH 6, PT link, DAKO). Specific preimmune sera or isotype‐specific unrelated primary antibodies were used for the control stainings. The detection system consisted of DAKO visualization reagent (dextran polymer conjugated with horseradish peroxidase and goat anti‐rabbit and antimouse immunoglobulins) with 3,3′‐ diaminobenzidine chromogen solution. Sections were counterstained with haematoxylin.

## 3 RESULTS

### 3.1 Histological characterization of GI wall diffuse stromal proliferation

**Figure 1:**
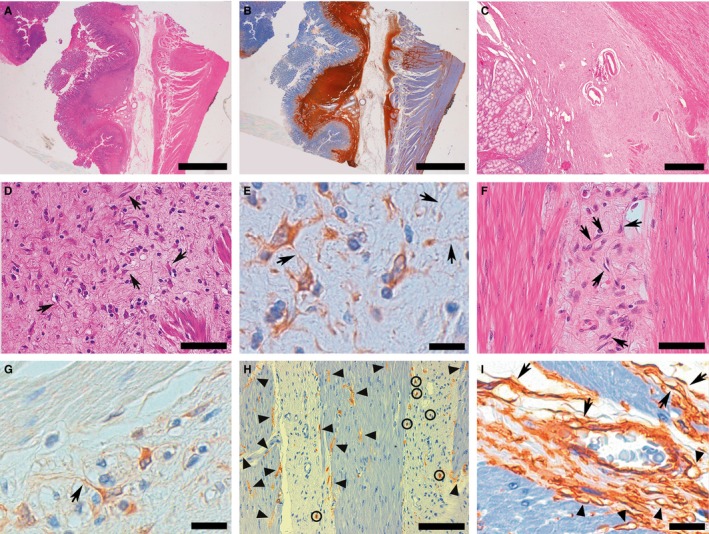
Representative examples of histological and immunohistochemical images of gastric TC hyperplasia found in *PDGFRA*‐mutant syndrome. A CD34+ stromal proliferation altered the structure of gastric wall, forming two thick bands along the inner and outer aspects of submucosa, sometimes forming ill‐defined nodules, and infiltrating the deeper wall layers with an interstitial pattern (A, H&E B, CD34). At times, the stromal proliferation obliterated submucosa (C). At higher magnification, the stromal proliferation consisted of spindle‐to‐polygonal PDGFRA+ TCs set in a variably collagenized background, often displaying slender, sometimes extremely long, cytoplasmic processes (telopodes), at times interconnecting with each other (arrows highlight some TCs whose telopodes are clearly evident due to the favourable intersection by the histological cutting surface) (D, H&E E, PDGFRA). The stromal proliferation with TCs (arrows, as in D and E) infiltrated muscularis propria with an interstitial pattern, separating smooth muscle fascicles, where no PDGFRA+ TCs are observed (F, H&E G, PDGFRA). CD117 demonstrated ICC‐IM, characteristically in close relation with smooth muscle fibres (arrowheads); mast cells (circled) were the only CD117+ cells populating the interstitial fibrosis (H). CD34 stained not only interstitial TCs (arrows), but also the endothelial lining of blood vessels: A small venule is evident at the centre of the picture; arrowheads indicate capillaries, identifiable because of their lumen (I). (Scale bars: A, B: 4 mm; C: 1 mm; D; 75 μm; E: 20 μm; F; 75 μm; G: 20 μm; H: 150 μm; I: 20 μm)

A proliferation of stromal tissue involved the stomach wall (Figure 1A) and proximal duodenum. It started as two bands lining the inner and outer aspects of the submucosa. These showed a tendency to merge and ultimately completely obliterate this structure (Figure 1C). Higher magnification revealed spindle‐to‐polygonal shaped cells set in a variably collagenized background, often displaying thin cytoplasmic processes, at times extremely long, at times apparently interconnecting (Figure 1D). When involving muscularis propria, they dissociated smooth muscle fascicles according to an interstitial pattern of growth (Figure 1F). These cells were diffusely and intensely positive for CD34 (Figure 1B and I) and PDGFRA (Figure 1E and G) but turned out to be CD 117 negative (Figure 1H). Besides the interstitial PDGFRA‐positive cells, CD34 IHC stained the endothelial lining of vessels, including the numerous capillaries present (Figure 1I). Conversely, CD117 revealed another population of cells with elongated cytoplasmic processes, not highlighted by PDGFRA IHC, either at the interface between fibrous tissue and smooth muscle fascicles or inside the latter, in close contact with smooth muscle cells; the only cell type stained in the fibrous intermuscular interstitium by CD117 IHC were mast cells (Figure 1H).

### 3.2 Relationship between GI wall diffuse stromal proliferation and IFP

**Figure 2:**
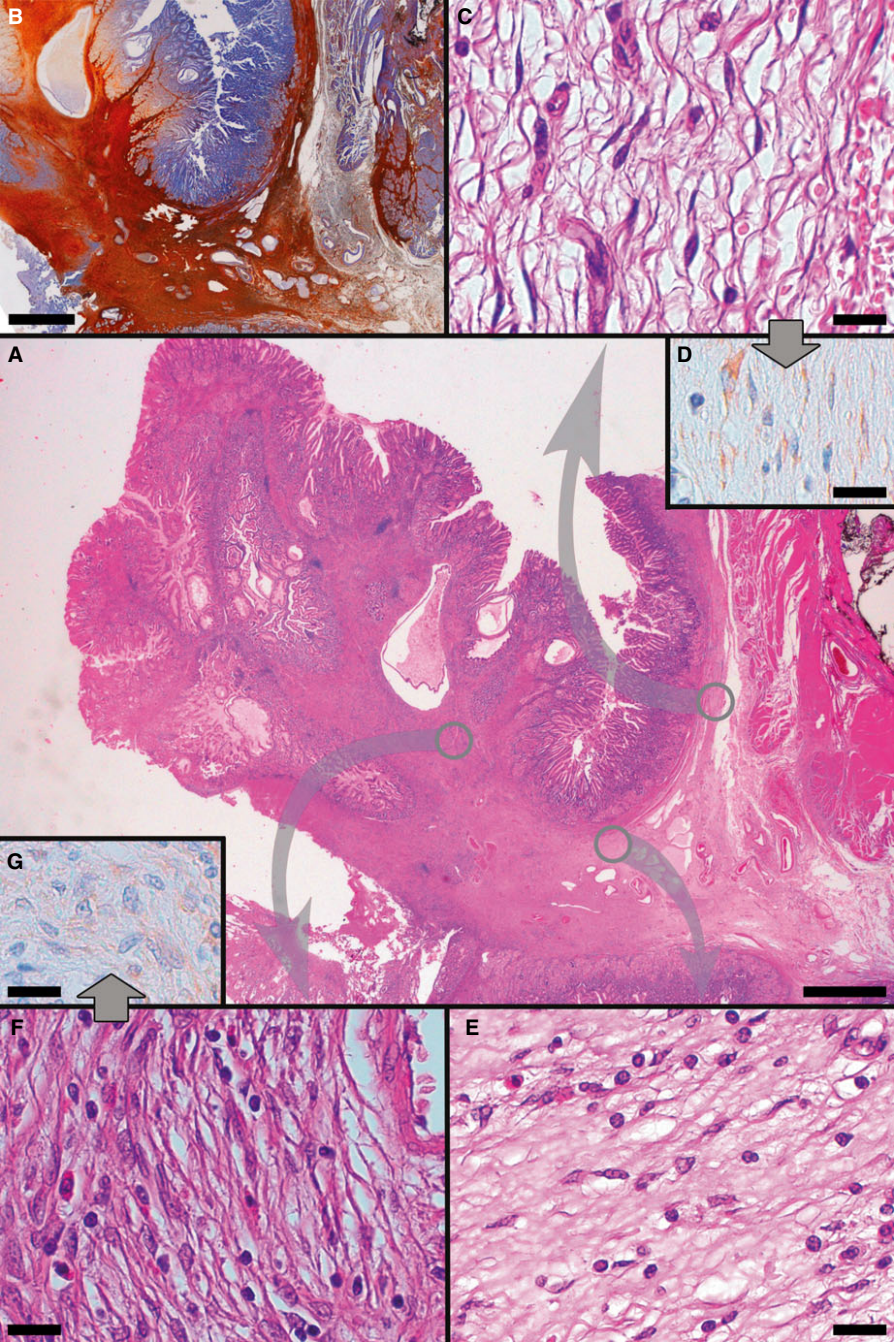
Representative examples of histological and immunohistochemical transition between TC hyperplasia and IFP found in *PDGFRA*‐mutant syndrome. At the centre, a panoramic view shows an IFP and its relation with TC hyperplasia (A, H&E). Proceeding in a clockwise direction, both TC hyperplasia and IFP stain positively at CD34 IHC (B). Diverse areas of the specimen are then shown, at higher magnification, in the following panels, which refer to areas encircled in grey in panel A; this is to underline the topographic location of the illustrated microscopic fields to better appreciate the transition between TC hyperplasia and IFP; corresponding areas are connected by arrows. Hyperplastic TCs featuring well‐developed, elongated telopodes, line the outer aspect of submucosa (C, H&E D, PDGFRA). Near the base of IFP, TCs tend to feature shorter cytoplasmic processes, or not to feature them at all (E, H&E). TCs eventually blur into the relatively plump mesenchymal cells characteristic of IFP (note the typical perivascular onion‐skin pattern and the presence of infiltrating eosinophils, absent in TC hyperplasia); PDGFRA positivity is maintained, albeit less intensely than in TC hyperplasia (F, H&E G, PDGFRA). (Scale bars: A, B: 2 mm; C‐G: 25 μm)

Several typical IFPs were detected, histologically superimposable to their sporadic counterpart, being composed of an admixture of fibroblast‐like CD34+ mesenchymal cells and inflammatory cells, often with numerous eosinophils, in a myxoid collagenous background. Interestingly, IFPs stemmed from the aforementioned diffuse stromal proliferation (Figure 2A and B) and a progressive loss of cytoplasmic processes in the constitutive cells was found at the interface of the two sectors, ultimately leading to the plump, spindled/polygonal/epithelioid stromal cells typical of PFI (Figure 2A,C,E,F). PDGFRA positivity was maintained, albeit with an overall decreased intensity (Figure 2D and G).

### 3.3 Relationship between GI wall diffuse stromal proliferation and GIST

**Figure 3:**
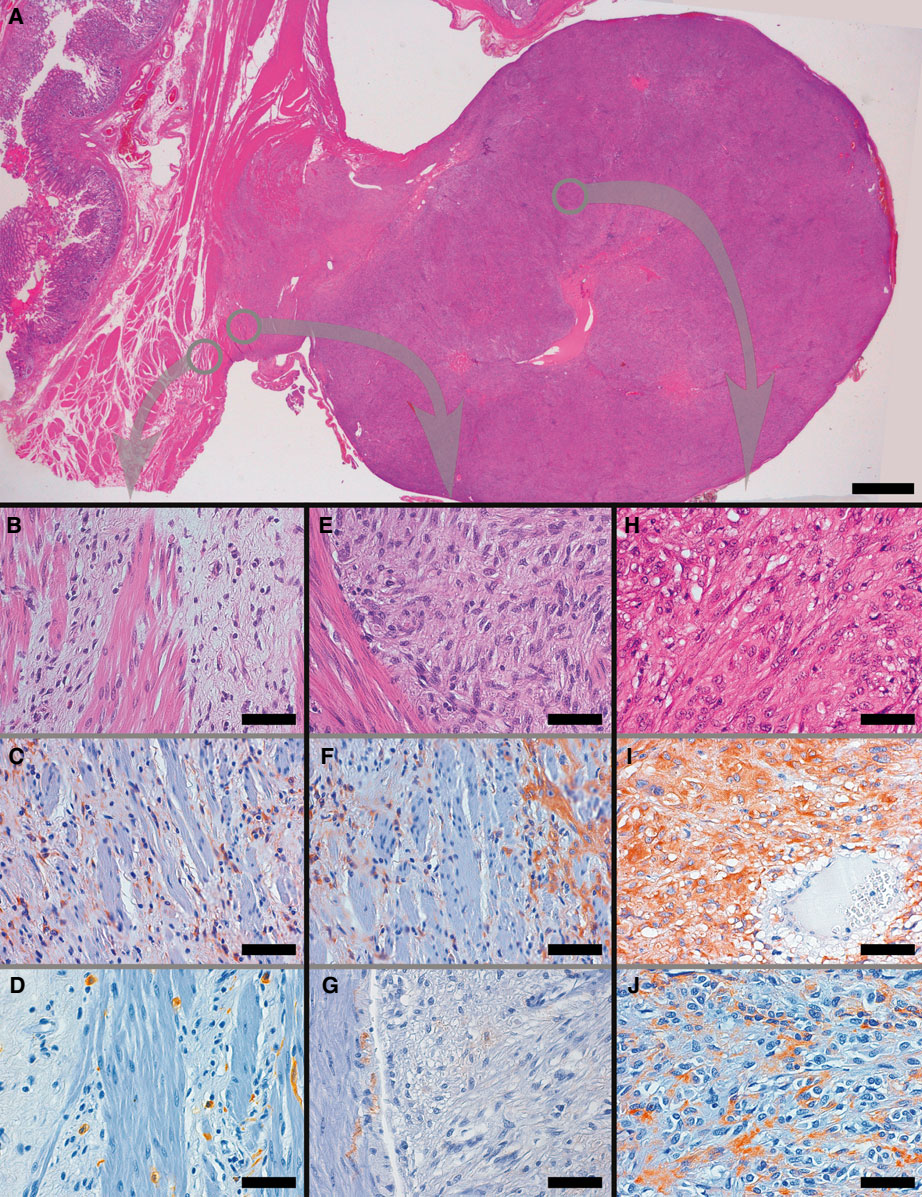
Representative examples of histological and immunohistochemical transition between TC hyperplasia and GIST found in *PDGFRA*‐mutant syndrome. The panoramic view shows a GIST originating from muscularis propria, whose smooth muscle bundles appear variously dissociated by the presence of interstitial TC hyperplasia, bulging on the external side of the gastric wall (A). As in Figure 2, higher magnification panels refer to areas encircled in grey in panel A, so as to underline the topographic location of the illustrated microscopic fields to better appreciate the transition between TC hyperplasia and GIST; corresponding areas are connected by arrows. Thin fibrous bands, populated by PDGFRA+ TCs, separate smooth muscle bundles near the periphery of GIST (B, H&E C, PDGFRA; D, CD117, highlighting ICC‐IM and mast cells). GIST (right) at the interface with muscularis propria (left) showed a relatively spindled cell cytology (E: H&E), and displayed an intense PDGFRA positivity (F), but only a weak and focal CD117 staining (G), thereby immunophenotypically resembling the neighbouring hyperplastic TCs rather than ICC‐IM. At a relative distance from the muscularis propria the GIST assumed a wholly epithelioid cytology (H, H&E), maintaining the intense PDGFRA immunoreactivity (I) and sometimes acquiring a comparatively strong positivity for CD117 (J). (Scale bars: A: 2 mm; B‐J: 65 μm)

Two gastric GISTs were present, whose histological features were indistinguishable from those of their sporadic *PDGFRA*‐mutant counterpart, featuring epithelioid cytology and PDGFRA and CD117 (patchy‐weak) positivity, as shown in Figure 3. Notably, near the gastric muscularis propria permeated by the previously described fibrous proliferation (Figure 3B) the GIST was characterized by a cytology that was relatively spindled (Figure 3E) compared to the overtly epithelioid type visible in the other parts of the tumour (Figure 3H). Moreover, unlike the nearby gastric wall, where both interstitial cells intensely positive for either PDGFRA or CD117 were present (Figure 3C‐D), the intramural periphery of GIST was intensely positive for PDGFRA only, with slightly focal, weak CD117 staining (Figure 3F‐G). A stronger, albeit patchy, CD117 positivity was present only towards the centre and in the subserosal portion of the GIST, accompanied by a persistent intense and diffuse PDGFRA reactivity (Figure 3I‐J).

## 4 DISCUSSION

We herein report a detailed morphological and immunophenotypical analysis of the diffuse stromal proliferation described in the GI wall in *PDGFRA*‐mutant syndrome^7^ and of its relationship with IFP and GIST, by exploiting the exceptional opportunity offered by its remarkable manifestation, together with the presence of these tumours, in an individual from a kindred previously published by the authors.^9^

As expected, germline *PDGFRA* mutations are able to originate multiple IFPs and GISTs.^7^ However, a third type of GI lesion, consisting of an intramural diffuse stromal proliferation variably referred to as “submucosal fibrous thickening,” “intestinal fibrosis,” “increased submucosal connective tissue,” “thickened submucosa” or “expanded submucosa”, has often been described in this genetic setting, both in humans and in mice.^8^, ^9^, ^10^ It consists of a CD34+ stromal proliferation, that variously involves the entire thickness of the GI tract, often heavily affecting the submucosa (Figure 1A‐C). In a previous paper describing *PDGFRA*‐mutant syndrome, we found this stromal proliferation remarkably evident in the stomach and duodenum of an individual, demonstrating that, in the submucosa, it often consisted of two thick bands running along its inner and outer limits; furthermore, its constitutive cells were positive not only for CD34, but also for PDGFRA, whereas both CD117 and DOG1 were negative.^9^ Here, besides confirming these characteristics, we show that these stromal cells often display long, slender cytoplasmic processes that sometimes appear to connect with each other (Figure 1D‐G). The consistent detection of these morphological features in cells positive at CD34 and at PDGFRA IHC (we did not perform a double immunostaining for CD34 and PDGFRA; this constitutes a limitation of our study) is coherent with the existence of a CD34/PDGFRA double‐positive cell type clearly distinct from other cells of the interstitium that can express PDGFRA, that is mast cells, or CD34, that is endothelial cells.^27^ In particular, endothelial lining often encircles vascular lumens, a feature allowing its clear identification without need of CD31 immunostaining, a useful additional tool for identifying endothelial cells in questionable cases.^28^ Thus, the stromal cells we describe display characteristics typical of TCs, both morphologically and immunophenotypically. In fact, TCs are characterized by a nucleated body (whose shape varies from small oval to triangular/polygonal or large round‐to‐oval, depending on the TCs’ anatomical location) that branches out into extremely long and thin prolongations, called “telopodes”; moreover, CD34 and PDGFRA are currently considered the most reliable markers for their immunohistochemical identification.^24^, ^29^ Additionally, the cells we describe are clearly distinguishable from ICCs because of both their immunophenotype and their compartmentalization. In fact, (i) they stain PDGFRA+ and CD117‐, unlike ICCs, which display a reciprocal staining; (ii) they recapitulate the physiological intramural distribution of TCs within the GI wall; (iii) in particular, they are present in the submucosa, where ICCs are not found 27 and, before completely obliterating it (Figure 1C), they form two thick bands along its inner and outer aspects (Figure 1A‐B), that is exactly where the TCs physiologically form a monolayer^24^; (iv) when in the muscularis propria, they are preferentially located within the interstitial “fibrosis,” whereas the CD117+ ICCs are found always in close contact with smooth muscle cells either at the interface between intermuscular stromal tissue and smooth muscle fascicles or within the latter (in keeping with intramuscular ICC‐ICC‐IM‐, known to be intercalated between the intramural neurons and smooth myocytes^30^) (Figure 1F‐H). Of note, nerve endings are rarely observed in the proximity of TCs.^24^ On the grounds of all these findings, it is our opinion that the GI wall diffuse stromal proliferation typical of *PDGFRA*‐mutant syndrome is sustained by hyperplastic TCs, constituting the “*PDGFRA*‐driven counterpart” of ICCH typical of germline *KIT* mutations. Of note, this finding confutes the histotype concealed by two of the terms previously used for defining the referred stromal proliferation, that is “submucosal fibrous thickening” and “intestinal fibrosis”: In fact, TCs are decreased in conditions determining a genuine fibrosis of GI wall such as Crohn's disease and ulcerative colitis.^28^, ^31^

We went on to ascertain whether it was possible to detect morphological and/or immunophenotypical clues potentially linking TC hyperplasia with IFP or GIST in *PDGFRA*‐mutant syndrome.

With regard to IFPs, we have already described examples stemming from the “submucosal thickening” found in an individual affected by *PDGFRA*‐mutant syndrome.^9^ Herein, we show also that there appears to be a gradual cytologic progression from TCs constituting the aforementioned thickening to stromal cells forming IFP, with a gradual loss of both slender cytoplasmic processes and relative cell elongation, accompanied by the acquisition of a relatively plumper morphology in the presence of a persisting, although overall less intense, positivity for PDGFRA (Figure 2C‐G). We therefore conclude that the TC hyperplasia forming the submucosal thickening characteristic of *PDGFRA*‐mutant syndrome, studied in this investigation, is pathogenetically related to IFP. Consequently, by analogy with what has been inferred from ICCH in germline *KIT*‐mutant settings regarding the relationship between ICC and *KIT*‐mutant GIST, TC can be considered the physiological counterpart of IFP. Consistently, it is noticeable that a subtype of TC, differing from the other GI TCs because of its lack of CD34 expression, has been described in the axes of intestinal villi both in humans and mice^27^ and that intestinal IFPs, unlike gastric ones, are often CD34‐.^1^, ^2^

Accumulating evidence has demonstrated that GISTs encompass a group of heterogeneous stromal tumours which, despite often sharing some features among overall GI location, spindle or epithelioid cytology, relatively mild atypia, low mitotic activity and immunoreactivity for DOG1 and CD117, nevertheless differ in molecular pathogenesis and, consequently, in anatomical distribution along GI tract, usual cytoarchitectural characters, immunophenotype, prognosis and drug sensitivity.^3^ Concerning the minority subgroup of *PDGFRA*‐mutant GISTs and their physiological counterpart, apart from the abovementioned lack of ICCH in *PDGFRA*‐mutant syndrome, it is worth noting that, unlike most other GISTs, these tumours are often CD117‐ or only weakly and patchy CD117+.^3^ Noticeably, TCs are CD117‐ in the GI tract, but may express this marker elsewhere.^24^, ^29^ Moreover, TCs are present within the muscularis propria (ie, the GI layer originating GISTs) both physiologically 24 and, with hyperplastic characteristics, in *PDGFRA*‐mutant syndrome (Figures 1 and 3). Under these circumstances, it is remarkable that an analysis of the transition between the gastric muscular wall and GIST in this latter condition demonstrates that (i) hyperplastic PDGFRA+ CD117‐ TCs are present in the muscularis propria close to the GISTs, along with physiological PDGFRA‐ CD117+ ICC‐IM (Figure 3A‐D); (ii) the periphery of the GIST close to muscularis propria shows a relatively spindled cytology together with an intense PDGFRA positivity but only a weak and focal CD117 staining, characteristics which, in relation to the two types of neighbouring interstitial cells, globally recall TCs rather than ICCs (Figure 3A,E‐G); (iii) a wholly epithelioid cytology is reached, sometimes flanked by a relatively strong CD117 immunoreactivity, only at a certain distance from muscularis propria (where PDGFRA positivity is nevertheless preserved) (Figure 3A,H‐J). Considering the hyperplastic condition of TCs in *PDGFRA*‐mutant syndrome, we believe that this evidence supports a relationship of TCs also with *PDGFRA*‐mutant GISTs.

Differences in TC features, possibly concealing different TC subpopulations and varying with both GI segment and GI wall layer,^24^ together with possible site‐specific differences in gene expression,^32^ could explain how TCs as a whole can originate diverse tumour types, and why, unlike IFPs, *PDGFRA*‐mutant GISTs show a strong (albeit not entirely exclusive) gastric predilection.^33^, ^34^

Our results are based on the study of the only individual featuring a prominent diffuse stromal proliferation of GI wall from a *PDGFRA*‐mutant kindred we previously investigated.^9^ In absolute terms, this constitutes a limitation to the significance of our conclusions. However, it must be considered that germline *PDGFRA* mutations are an exceedingly rare event in humans, with only five to six examples (considering kindreds or single individuals) reported in the literature^7^, ^8^; on top of that, the presence of GI diffuse stromal proliferation together with IFPs and/or GISTs, that is the ideal setting for investigating the relationship between these latter tumours and TCs, has been reported in a fraction only of mutants (in particular, with regard to the kindred we previously published, only in the proband, that is member II‐2‐).^8^, ^9^ The study of sporadic IFPs and *PDGFRA*‐mutant GISTs is probably of limited utility for defining a possible link between these tumours and TCs as, under these circumstances, only the fully developed neoplastic morphological and immunophenotypical characters of the former tumours are observable, features which could be deemed not to be specific enough in terms of cell lineage in the absence of any type of transition with respect to physiological TCs

In conclusion, our results support TCs as the physiological counterpart of both IFPs and *PDGFRA*‐mutant GISTs, possibly pathogenetically related to both of these tumour types. Furthermore, on the basis of our results we herein propose “telocytoma” as a more appropriate term than IFP for defining the lesion formerly referred to as “Vanek's tumour.” In fact, compared to the misleading “inflammatory fibroid polyp” definition that evokes an aspecific inflammatory‐reactive lesion, “telocytoma” conveys both the correct pathogenetic (that is neoplastic) and histotypic (that is “telocytary”) essence of this lesion (semantically reiterating the changeover from “colonic fibroblastic polyp” to “colonic perineurioma” 35). Further studies are warranted to further shed light on this fascinating topic.

## CONFLICTS OF INTEREST

Riccardo Ricci declares speaker's honoraria from Novartis; the other authors confirm that there are no conflict of interests.
